# MCRS2 represses the transactivation activities of Nrf1

**DOI:** 10.1186/1471-2121-10-9

**Published:** 2009-02-02

**Authors:** Jia-Long Wu, Young-Sun Lin, Chi-Chiang Yang, Yu-Jen Lin, Shan-Fu Wu, Ying-Ting Lin, Chien-Fu Huang

**Affiliations:** 1 IBMS, Academia Sinica, Taipei 11529, Taiwan, R.O.C; 2 Development Center for Biotechnology, Taipei 11529, Taiwan, R.O.C; 3 School of Medical Laboratory and Biotechnology, Chung Shan Medical University, Taichung, Taiwan, R.O.C; 4 Department of Fashion Design & Management, Tainan University of Technology, Tainan, Taiwan, R.O.C; 5 Department of Biotechnology, College of Life Sciences, Kaohsiung Medical University, Kaohsiung, Taiwan, R.O.C; 6 Department of Biological Science and Technology, I-Shou University, Kaohsiung, Taiwan, R.O.C

## Abstract

**Background:**

Nrf1 [p45 nuclear factor-erythroid 2 (p45 NF-E2)-related factor 1], a member of the CNC-bZIP (CNC basic region leucine zipper) family, is known to be a transcriptional activator by dimerization with distinct partners, such as Maf, FosB, c-Jun, JunD, etc. The transcriptional roles of CNC-bZIP family are demonstrated to be involved in globin gene expression as well as the antioxidant response. For example, CNC-bZIP factors can regulate the expression of detoxification proteins through AREs, such as expression of human gamma-glutamylcysteine synthetases (GCS), glutathione S-transferases (GST), UDP-glucuronosyl transferase (UDP-GT), NADP (H) quinone oxidoreductase (NQOs), etc. To further explore other factor(s) in cells related to the function of Nrf1, we performed a yeast two-hybrid screening assay to identify any Nrf1-interacting proteins. In this study, we isolated a cDNA encoding residues 126–475 of MCRS2 from the HeLa cell cDNA library. Some functions of MCRS1 and its splice variant-MSP58 and MCRS2 have been previously identified, such as transforming, nucleolar sequestration, ribosomal gene regulation, telomerase inhibition activities, etc. Here, we demonstrated MCRS2 can function as a repressor on the Nrf1-mediated transactivation using both in vitro and in vivo systems.

**Results:**

To find other proteins interacting with the CNC bZIP domain of Nrf1, the CNC-bZIP region of Nrf1 was used as a bait in a yeast two-hybrid screening assay. MCRS2, a splicing variant of p78/MCRS1, was isolated as the Nrf1-interacting partner from the screenings. The interaction between Nrf1 and MCRS2 was confirmed *in vitro *by GST pull-down assays and *in vivo *by co-immunoprecipitation. Further, the Nrf1-MCRS2 interaction domains were mapped to the residues 354–447 of Nrf1 as well as the residues 314–475 of MCRS2 respectively, by yeast two-hybrid and GST pull-down assays.  By immunofluorescence, MCRS2-FLAG was shown to colocalize with HA-Nrf1 in the nucleus and didn't result in the redistribution of Nrf1. This suggested the existence of Nrf1-MCRS2 complex in vivo. To further confirm the biological function, a reporter driven by CNC-bZIP protein binding sites was also shown to be repressed by MCRS2 in a transient transfection assay. An artificial reporter gene activated by LexA-Nrf1 was also specifically repressed by MCRS2.

**Conclusion:**

From the results, we showed MCRS2, a new Nrf1-interacting protein, has a repression effect on Nrf1-mediated transcriptional activation. This was the first ever identified repressor protein related to Nrf1 transactivation.

## Background

Nrf1 (NF-E2 related factor1) belongs to the Cap'n'Collar-basic leucine zipper proteins (CNC-bZIP). The CNC-bZIP family is identified by its homology region, named the CNC domain, immediately N-terminal to the bZIP domain [1]. This family contains a basic domain interacting with sequence-specific DNA and a leucine zipper domain (bZIP) involved in protein-protein dimerization [2-4]. The members of Cap'n'Collar (CNC) family contain Nrf1, Nrf2, Nrf3, p45 NF-E2, *Drosophila *CNC protein, as well as *C. elegans *Skn-1 [5-9]. There are two known transcriptional roles of the CNC-bZIP family. First, they are involved in globin gene expression. Transcriptional control of the human beta-globin gene cluster is mediated by four DNase I hypersensitive sites (HS1–4) spanning approximately 6–22 kb upstream of the epsilon-globin gene [10-13]. A similar gene structure (HS-40) is also present in alpha-globin gene expression [10,14,15]. The locus control region (LCR) appears to be necessary for high protein level expression from the entire globin gene clusters. The LCRs contain a direct sequence repeats 5'-(A/G)TGA(C/G)TCAGC(A/G)-3', which is the binding site for CNC-bZIP transcription factors [16]. Second, they play a role in the antioxidant response. The consensus antioxidant response element (ARE) core sequence 5'-TGA(C/G)NNNGC-3' shows remarkable similarity to the aforementioned binding sequence for CNC-bZIP proteins. This similarity has led to the proposal CNC-bZIP factors can regulate detoxification proteins expression through AREs [17-21], such as expression of human gamma-glutamylcysteine synthetases (GCS), glutathione S-transferases (GST), UDP-glucuronosyl transferase (UDP-GT), NADP (H) quinone oxidoreductase (NQOs), etc.

Like the other members of CNC-bZIP factors, Nrf1 can heterodimerize with small Maf proteins to bind the cis-elements more efficiently [16,22]. The small Maf proteins, including MafK, MafG and MafF, are identified by their homology to the avian transforming retroviral oncogene, v-maf with lacking of transactivation domains [23-25]. Transcriptional repression by Maf-Maf homodimers, yet activation by Maf-Nrf heterodimers, is shown in LCR-regulated gene regions [22]. To further explore other factor(s) in cells related to Nrf1, we performed a yeast two-hybrid screening assay using the CNC-bZIP region of Nrf1 as a bait to identify any Nrf1-interacting proteins. In this study, cDNAs encoding residues 126–475 of MCRS2 (or residues 185–534 of p78/MCRS1, or residues 113–462 of MSP58 [26-28] were isolated from the HeLa cell cDNA library. In this paper, we characterized the physical and functional interactions between Nrf1 and MCRS2.

Some functions of MCRS1 and its splice variant-MSP58 and MCRS2 have been reported before, such as transforming, nucleolar sequestration, ribosomal gene regulation, and telomerase inhibition activities [28-32]. For example, TOJ3, a protein with high structural similarity to MCRS1, display transformation activity [32]. In addition, the tumor suppressor gene PTEN suppresses the transformation activity of MCRS1 [30]. It was reported MSP58 can relieve the repressor activity of Daxx, an adaptor protein that links Fas signaling to the c-Jun NH2-terminal kinase pathway by a nucleolar sequestration mechanism [31]. Further, MCRS2 is also shown to be involved in telomere shortening by interacting with telomerase [28]. Although these studies illustrates several functions of MCRS1/p78 and its splice variant, the roles of MCRS2 on Nrf1 transactivation activity have not been previously identified. Here we will show MCRS2 can function as a repressor on the Nrf1-mediated transactivation.

## Results

### Identification of MCRS2 as a Nrf1-interacting protein

**Figure 1:**
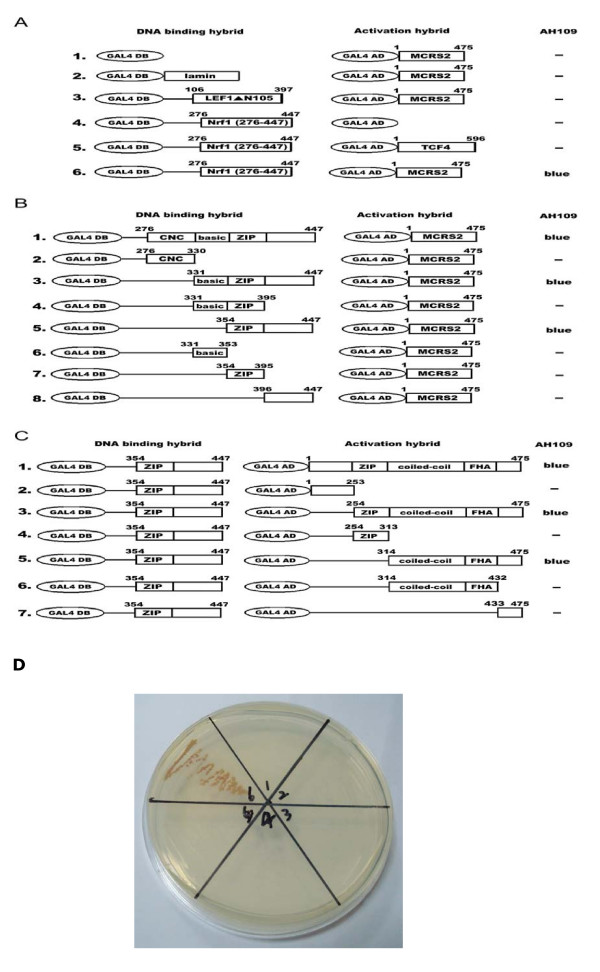
**Interaction of MCRS2 with Nrf1**. *Left column*, GAL4 DNA binding domain (amino acids 1–147) hybrids. *Middle column*, GAL4 activation domain (amino acids 768–881) hybrids. *Right column*, yeast colony color after transformation. Minus (-) indicated no yeast colony was noted following growth incubation. **A**, characterization of the interaction between Nrf1 and MCRS2. *Left column*, the GAL4 DNA-binding domain (GAL4BD) hybrid. *Middle column*, the GAL4 DNA-activation domain (GAL4AD) hybrid.* Right column*, yeast colony color after transformation. The lamin, LEF1ΔN105, and TCF4 constructs in this experiment were used for the negative control. **B**, The C terminus of Nrf1 was required and sufficient for the interaction with MCRS2. *Left column*, the various GAL4DB-Nrf1 deletion derivatives. *Middle column*, GAL4AD-MCRS2.* Right column*, yeast colony color after transformation. The Nrf1 fragments fused to the GAL4 DNA binding domain are indicated on the *left *of the diagrams. The relative positions of CNC, basic, and ZIP domains of Nrf1 are also depicted. **C**, The C terminus of MCRS2 was required and sufficient for the interaction with Nrf1.* Left column*, GAL4DB-Nrf1 (354–477).* Middle column*, the various GAL4AD-MCRS2 deletion derivatives, *Right column*, yeast colony color after transformation. The MCRS2 fragments fused to GAL4 activation domain are shown in the *middle *of the diagrams. The relative positions of putative ZIP, coiled-coil, FHA domains of MCRS2 are also depicted. **D**, The photograph of colony formation came from the results of A. The labels of Figure 1D were the same labels of A.

In searching for proteins specifically interacting with the important domain of Nrf1, we screened a *HeLa *MATCHMAKER activation domain library (Clontech) using a chimeric protein construct composed of the CNC-bZIP domain (*residues 276–447*) of Nrf1 fused to GAL4 DNA binding domain as a bait [GAL4DB-Nrf1(276–447)]. Fusion protein GAL4DB-lamin and GAL4DB-Lef1▲N105 were also included in assays to remove possible false positive clones. Roughly 10^6 ^colonies were screened with this yeast two-hybrid system. Among the clones identified and sequenced, a few clones contained cDNA encoding the C-terminal region of either MSP58 protein (*residues 113–462*), p78/MCRS1 (*residues 185–534*), or MCRS2 (*residues 126–475*) [28]. To examine whether Nrf1 interacted with full-length MCRS2, the interaction between Nrf1 and MCRS2 was also confirmed in a yeast two hybrid system. As summarized in Fig. 1A, MCRS2, consistent with the data obtained with truncated versions of MCRS2, interacted quite well with Nrf1 (276–447) (*row 6*). Control experiments confirmed the specificity of the Nrf1-MCRS2 interactions: introducing plasmid pGAL4DB-Nrf1 (276–447) alone into yeast produced no colony (*row 4*). Similarly, no colony was observed upon co-transformation of pGAL4DB-Nrf1 (276–447) with pGAL4AD-TCF4 (*row 5*), which was the negative control. Thus, two conclusions could be drawn from the above experiments. First, there was a specific interaction between Nrf1 and MCRS2. Second, the bZIP to C-terminus domain of Nrf1 was involved in the binding to MCRS2.

### Mapping the interaction domain between MCRS2 and Nrf1

Because MCRS2 displayed relatively high affinity toward Nrf1, we set out to map the MCRS2-binding domain of Nrf1 with a yeast two-hybrid system using GAL4AD-MCRS2 and a panel of GAL4 DB-Nrf1 deletion constructs. As summarized in Fig. 1B, the residues 276–447 of Nrf1 interact with MCRS2 (*row 1*). The CNC region of Nrf1 (*residues 276–330*) failed to interact with MCRS2 (*row 2*). While *residue 331–447 *of Nrf1, composed of the basic region, the ZIP region and the most C-terminal region contributed to the binding of MCRS2 (*row 3*). Thus, *residues 354–447 *of Nrf1, including the zipper region and the most C-terminal region, constituted the minimal MCRS2-binding domain (compare *row 3 *and *5 *to *row 4*, *6*, *7 *and *8*). These findings suggest the CNC region (compare *row 1 *to *row 2 *and *3*) and basic region (compare *row 3 *to *row 5 *and *6*) of Nrf1 were not required to interact with MCRS2. Interestingly, although the *residues 396–447 *of Nrf1 encompassing the region C-terminal to the zipper of Nrf1 didn't bind directly to MCRS2 (*row 8*), they were absolutely required for the interaction between Nrf1 and MCRS2 (compare *row 5 *to *row 7 *and *8*).

We also performed mapping of Nrf1-binding domain of MCRS2 by yeast two-hybrid assay using a panel of various deletion constructs of GAL4AD-MCRS2. As shown in Fig. 1C, the minimal MCRS2-interacting domain of Nrf1 (*residues 354–447*) interacted with MCRS2 (*row1*) and C-terminal region of MCRS2 spanning residue 254–475 (contain putative ZIP domain, coiled-coil domain, FHA domain, and the most C-terminal region) interacted with Nrf1 (*row 3*). The N-terminal region of MCRS2 (*residues 1–253*) failed to interact with Nrf1 (*row 2*). Further, the minimal Nrf1-interacting domain of MCRS2 was mapped to the residues 314–475 (contain the coiled-coil domain, FHA, and the most C-terminal reagion), and the C-terminus of MCRS2 (*residues 433–475*) was required for Nrf1-MCRS2 interaction (*row 5–7*). Taken together, we concluded the coiled-coil and FHA domain of MCRS2 were indispensable for its interaction with Nrf1.

### Interaction of Nrf1 with MCRS2 *in vitro *and *in vivo*

**Figure 2:**
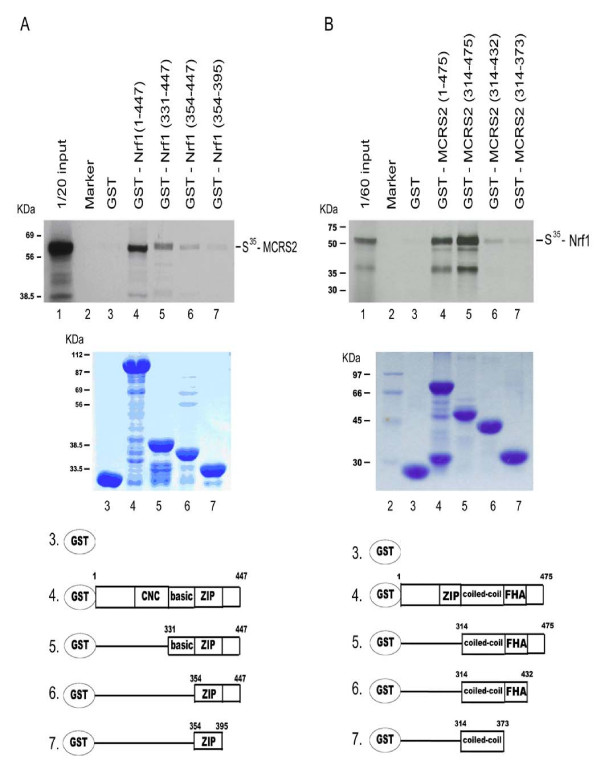
***In vitro *direct interaction of Nrf1 with MCRS2**. **A**, binding of MCRS2 by various GST-Nrf1 deletion mutants is shown. The GST protein bead used is indicated *above *for each track of the autoradiogram. *Top panel*, *in vitro *translated MCRS2 was incubated with GST (*lane 3*) or GST-Nrf1 derivatives (*lanes 4–7*) beads at 4°C for 2 hours and then was eluted with 0.1 M reduced glutathione. The autoradiography from the eluted supernatants was separated on a 10% SDS-PAGE gel. *Lane 1 *indicates 1/20 labelled MCRS2 (S^35^-MCRS2). *Middle panel*, the same amounts of purified GST (*lane 3*) and GST-Nrf1 derivatives (*lanes 4–7*) were used in a GST pull-down assay, separated on a 10% SDS-PAGE gel, and then stained. *Bottom panel*, diagram of the purified GST and GST-Nrf1 derivatives. **B**, binding of Nrf1 by various GST- MCRS2 deletion mutants is shown. The GST protein bead used is indicated *above *each track of the autoradiogram. *Top panel*, *in vitro *translated Nrf1 was incubated with GST (*lane 3*) or GST-MCRS2 derivatives (*lanes 4–7*) beads at 4°C for 2 hours and was then eluted with 0.1 M reduced glutathione. The autoradiography from the eluted supernatants was separated on a 10% SDS-PAGE gel. *Lane 1 *indicated 1/60 labelled Nrf1 (S^35^-Nrf1). *Middle panel*, the same amounts of purified GST (*lane 3*) and GST-MCRS2 derivatives (*lanes 4–7*) were used in GST pull-down assay, separated on a 10% SDS-PAGE gel, and then stained. *Bottom panel*, diagram of the purified GST and GST-MCRS2 derivatives.

To test whether Nrf1 interacted with MCRS2 *in vitro*, various affinity matrixes consisting of various deletion constructs of GST-Nrf1 or GST-MCRS2 fusion proteins as the ligands were prepared. The S^35^-labled full-length protein of either Nrf1 or MCRS2 was incubated with the matrix, and the bound proteins were analyzed by SDS-PAGE. Fig. 2A shows, consistent with data obtained with the yeast two-hybrid system (Fig. 1), MCRS2 (*lane 4–6*) bound to GST-Nrf1 (1–447), GST-Nrf1 (331–447), and GST-Nrf1 (354–447). However, GST *(lane 3*) and GST-Nrf1 (354–395) (*lane 7*) failed to be retained under the same experimental conditions. Moreover, Nrf1 (331–447) had higher affinity toward MCRS2 than Nrf1 (354–447) did (compare *lane 5 *with *lane 6*). The data agree with the results obtained from the yeast two-hybrid experiments (Fig. 1).

In addition, the interactions between S^35^-labled Nrf1 and various deletion constructs of GST-MCRS2 fusion protein were also demonstrated in the same way (Fig. 2B). Nrf1 failed to interact with GST (*lane 3*) and GST-MCRS2 (314–373) (*lane 7*). In contrast, Nrf1 interacted with full-length MCRS2 and C-terminal of MCRS2 (*residues 314–475*) (*lane 4 and lane 5*). Then S^35^-Nrf1 showed a decreasing affinity toward the C-terminal region of MCRS2 (*lane 5–7*). The results were consistent with the data obtained from yeast two hybrid results (Fig. 2B).

**Figure 3:**
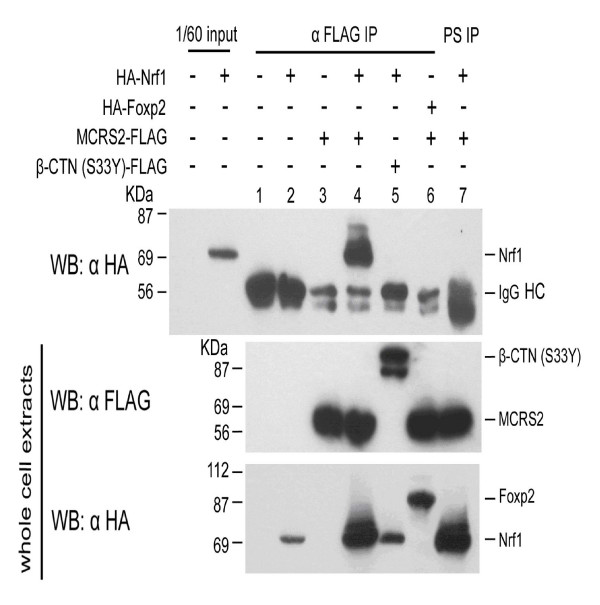
**Nrf1 interacts with MCRS2 *in vivo***. *Top row*, the transfected expression plasmids are indicated. Total cell extracts were prepared from the cells transfected with the indicated expression plasmids and were then incubated with an antibody against FLAG epitope (*lane 1–6*) or a pre-immune serum (*lane 7*). The resulting co-immunoprecipitates were separated on a 10% SDS-PAGE gel and probed with an antibody against HA epitope. The control experiments (*middle and bottom rows*) were done using the same cell extracts as the *top row *to indicate the transfected cell indeed express FLAG fusion protein (*middle row*) and HA fusion protein (*bottom row*) and were detected by anti-FLAG or anti-HA antibody, respectively. *IgG HC*, immunoglobulin heavy chain. *WB*, western blot. *PS*, pre-immune serum.

To determine whether MCRS2 associates with Nrf1 in mammalian cells, plasmids expressing MCRS2-FLAG and HA-Nrf1 fusion protein were co-transfected into 293 cells for co-immunoprecipitation assay study. Cell extracts were prepared from transfected cells and then immunoprecipitated with an antibody against FLAG. As summarized in Fig. 3, Nrf1 only co-precipitated in the presence of MCRS2 by an anti-FLAG antibody but not in the presence of a preimmune serum (*top panel, compare lane 4 with lane 7*). No Nrf1 co-precipitation was noted when cells overexpressed Nrf1 plus Nrf1-unrelated protein β-catenin (S33Y) (*lane 5*). No foxp2 co-precipitation was observed when cells overexpressed MCRS2 and unrelated Foxp2 (*lane 6*). From this data, we concluded Nrf1 and MCRS2 formed a complex *in vivo*.

### Colocalization of Nrf1 and MCRS2 in the nucleus

**Figure 4:**
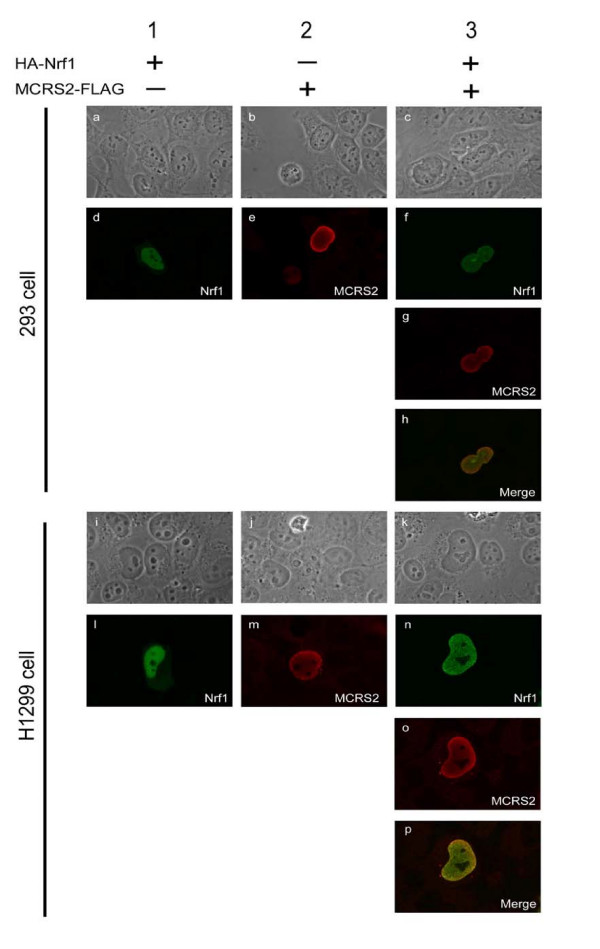
**Co-localozation of Nrf1 and MCRS2**. 293 (*panel a-h*) and H1299 cells (*panel i-p*) were transfected with plasmid constructs expressing either HA-Nrf1 alone (*panel a, d, i and l*), MCRS2-FLAG alone (*panel b, e, j and m*), or HA-Nrf1 and MCRS2-FLAG together (*panel c, f, g, h, k, n, o and p*). After 24 hours, cells were fixed and stained with the antibodies. The antibodies used were the M2 monoclonal antibody against FLAG-tagged MCRS2 and the rabbit polyclonal anti-HA antibody against the HA-tagged Nrf1. The cells were viewed with the phase-contrast microscope (*panel a-c and i-k*). The green signal (αHA-Nrf1) was obtained with a FITC-conjugated secondary antibody under a confocal microscope (*panel d, f, l and n*). The red signal (αMCRS2-FLAG) was obtained with a Rhodamin-conjugated secondary antibody under confocal microscope (*panel e, g, m and o*). The merged images (*panel h and p*) showed the co-localization of Nrf1 and MCRS2.

We attempted to correlate our findings of a Nrf1-MCRS2 complex in living cells by analyzing the subcellular distribution of these two proteins. In previous studies, exogenously expressed Nrf1 was found to be localized chiefly in the nucleus [16]. MCRS2 was also reported to be co-localized with LTPS/PinX1 in the nucleus, especially in nucleoli [28]. We used the HA-Nrf1 and MCRS2-FLAG fusion proteins as well as corresponding fluorescent antibodies as the dynamic fluorescent markers to facilitate detection of the two partner's subcellular distribution (Fig. 4). Immunofluorescence detection of HA-Nrf1 (green) and MCRS2-FLAG (red) was obtained by rabbit anti-HA and mouse anti-FLAG antibodies and followed by the corresponding species-specific labelled antibody. In our study, Nrf1 was colocalized with MCRS2 in the nucleus (Fig. 4, panel h and p). Co-expression of Nrf1 and MCRS2 did not cause cellular redistribution of these two proteins (compare *lane 1–2 *with *lane 3*). These results taken together strongly suggest Nrf1-MCRS2 complex can exist *in vivo *and MCRS2 might participate physiologically in Nrf1-mediated transcriptional activity.

### Repression of transactivational activity of Nrf1 by MCRS2

**Figure 5:**
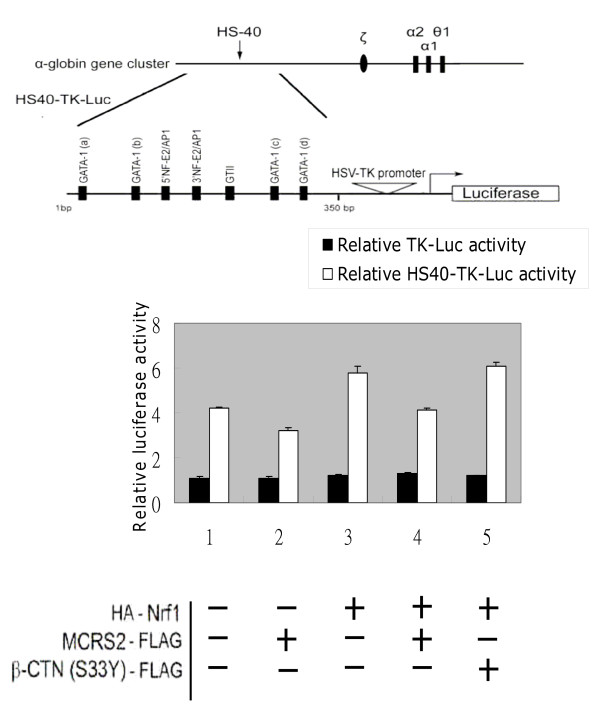
**Transcription driven by NF-E2 binding sites is repressed by MCRS2**. Transient transfections were performed in 293 cells using HS40-TK-Luc as a reporter. HS40-TK-Luc was constructed by inserting the HS40 element containing NF-E2 binding sites before the HSV-TK promoter of TK-Luc. Briefly, the cells were transiently transfected with luciferase reporter (0.2 μg), β-galactosidase reporter (0.05 μg), and the plasmid constructs expressing HA-Nrf1 (0.2 μg), MCRS2-FLAG (0.4 μg) and β-catenin (S33Y)-FLAG (0.4 μg) as indicated. 36 hours after transfection, the activities of luciferase and β-galactosidase were determined as described under "Experimental procedures". Luciferase activity from transfected cells was normalized with the β-galactosidase activity and expressed as relative luciferase activities compared with the control (*lane 1, black bar*). Error bars represent the mean ± S.D. from three independent determinations. β-catenin (S33Y)-FLAG in this experiment was used as the control for comparing the effects from MCRS2-FLAG. Other control experiments were also preceded as above, except the reporter was only driven by the HSV-TK promoter (*black bar*).

**Figure 6:**
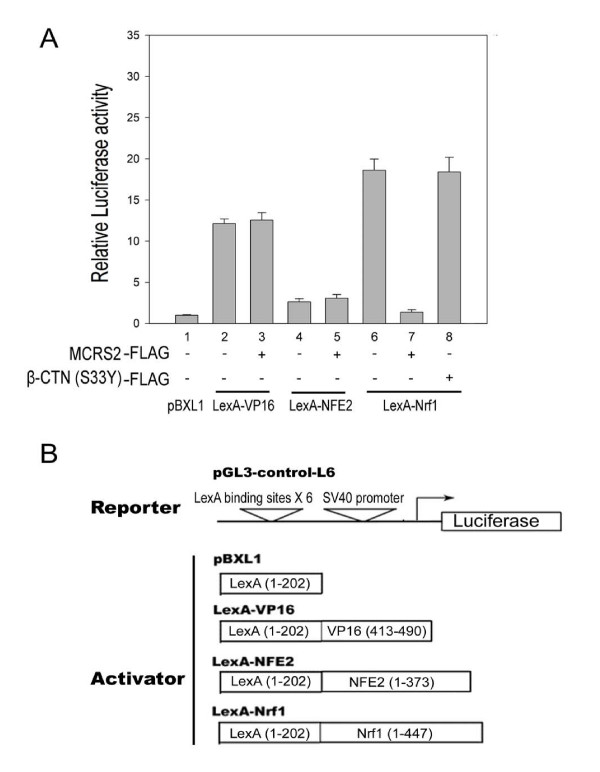
**Nrf1-mediated transcriptional activation is repressed by MCRS2**. **A**, MCRS2 mediates transcriptional repression on the transcriptional activity of LexA-Nrf1. Transient transfections were performed in 293 T cell using pGL3-control-L6 as the reporter containing six LexA-binding sites. The LexA-bZIP family fusion proteins and MCRS2 in each transfection are indicated below each track of the activities. Briefly, cells were transfected with pGL3-control-L6 reporter (0.2 μg), β-galactosidase reporter (0.1 μg), and either plasmid constructs expressing LexA (0.6 μg), LexA-Vp16 (0.6 μg), LexA-NFE2 (0.6 μg), or LexA-Nrf1 (0.6 μg) with or without MCRS2 (0.5 μg) or β-catenin (S33Y) (0.5 μg) as indicated. 36 hours after transfection, the activity of luciferase and β-galactosidase were determined as described under "Experimental procedures". In each experiment, luciferase activity from transfected cells was normalized with the β-galactosidase activity and expressed as relative luciferase activities compared with the control (LexA only, *lane 1*). The error bar represents the mean ± S.D. from three independent experiments. **B**, diagrams of the reporter and the activator structures.

Because Nrf1 interacted with MCRS2 *in vivo *(Fig. 3 and Fig. 4), it was important to ask whether the transcriptional activity of Nrf1 was modulated by MCRS2. To address this question, two complementary experiments were performed. First, a reporter activity driven by HS40 element of α-globin Locus Control Region, containing the CNC-bZIP protein binding sites, was measured under the influence of MCRS2. As illustrated in Fig. 5, transcription of the reporter, HS40-TK-Luc, was inhibited by MCRS2 but not by another unrelated protein β-catenin (S33Y) (compare *lane 2–4, white bar*). Substitution of the Nrf1 binding sites with the minimal TK promoter rendered the resultant reporter, TK-Luc, unresponsive to MCRS2 (compare *lane 3 *and *lane 4*, *black bar*). We concluded the MCRS2-mediated repression depended on the presence of the NF-E2 binding sites (compare *black bar *with *white bar*). Second, because multiple CNC-bZIP factors can interact with the NF-E2 site, the identity of CNC-bZIP factors involved in the repression was determined by measuring the effect of MCS2 on the transcription activities of LexA-CNCbZIP fusion proteins. As shown in Fig. 6, the transcriptional activity of the LexA-Nrf1 was largely reduced in the presence of MCRS2 in 293 T cells (compare *lanes 6*-*7*). The reduction was specific, because MCRS2 had little effect on LexA-VP16 and LexA-NFE2. Based on data obtained from the aforementioned experiments, we believe a direct interaction between Nrf1 and MCRS2 was required for the observed specific repression.

## Discussion

Based on the results from yeast two-hybrid and immunoprecipitation assays presented here, we believe MCRS2 interacted with Nrf1 and suppressed Nrf1-mediated transcriptional activity. Since Nrf1-mediated transactivation plays an important role in antioxidant response and embryo development, its activities need to be well controlled [17,18,20,33,34]. Small Maf protein is a well-known interaction partner of Nrf1. Nrf1/Maf heterodimer recognizes the NF-E2/AP1-like motif and activates downstream gene expression [16,22]. Other Nrf1-interacting proteins such as AP1 proteins (c-Jun, JunB, and JunD) also regulate antioxidant response element (ARE)-mediated detoxifying enzymes expression in response to antioxidants and xenobiotics [17]. Until now there has never been any report of a protein repressing the Nrf1-mediated transactivation. Here, we found MCRS2 has a high tendency to repress Nrf1-mediated transactivation.

CNC-bZIP factors binding site is also reportedly involved in the transcriptional regulation of many cellular protective and antioxidant genes [17-21]. Thus, by interacting with Nrf1, MCRS2 may shift the balance between activation and repression of these regulatory genes and result in the modulation such as detoxifying mechanisms. In fact, we believe the promoters of NQO1 and other detoxifying enzyme genes are possibly regulated by Nrf1/MafG; and the differential effects of Nrf2 and Nrf1 with small Maf proteins on NQO1 gene activation remain unknown. Therefore, the unpredictable interaction between MCRS2 and Nrf1 may play some role in the upper issue.

The c-terminus (719–772) of the long Nrf1 behind the zipper domain is the Neh3 domain of Nrf1. This domain is required but not sufficient for interaction with MCRS2 (containing coiled-coil domain, FHA domain, and C-terminus) in the yeast two-hybrid system. The specific interaction between MCRS2 and the Neh3 domain appears to be important for the interaction with Nrf1 and suggests Neh3 of Nrf1 may be involved in recruiting accessory proteins or serve to bridge the transcription factor to the active transcription apparatus [35]. The precise mechanism of the interactions between Neh3 of Nrf2 and CHD6 leading to gene transcription has been demonstrated but is not fully understood by a previous study (Nioi et al, 2005). The findings of that paper provide strong evidence suggesting the Neh3 domain may act as a TAD. The activity of the Neh3 domain relies on the VFLVPK motif, which is chiefly hydrophobic in nature and could be a site of interaction with a novel partner molecule. Further, sequences from N terminal to the VFLVPK motif of Neh3 domains are also very highly conserved among the CNC-bZIP family and critical for the interaction of Nrf1 with MCRS2. The interaction of MCRS2 with other members of the CNC-bZIP family needs to be further explored. The critical F591 residue of Nrf2 Neh3 domain is changed to L748 residue on Nrf1. It is worth exploring whether this change influences the interaction between Nrf1 and CHD6 or determines the different interactions of Nrf1 with MCRS2 and CHD6. The Neh3 domain functions as a protein-protein interaction module to facilitate the recruitment of putative coactivator proteins, such as MCRS2, to Nrf1-dependent genes. This phenomenon is similar to the interaction between CHD6 and Nrf2, but MCRS2 negatively regulates Nrf1, while CHD6 positively regulated Nrf2. It is an interesting question of whether Nrf1 could be regulated by both MCRS2 and CHD6 and if the contrast effects of MCRS2 and CHD6 influence Nrf1-mediated ARE-driven gene expression.

The Nrf1 protein utilized in this study is slightly shorter than TCF11 and contains 447 amino acids without its N-terminal domain. The protein was obtained by alternative splicing of mRNA. In particular, the peptide sequence GLLQFTILLSLIGVRVD termed the NHB1 (N-terminal homology box 1) found between residues 11 and 27 of Nrf1 shares 35% of its identity with the MLLSVPLLLGLLGLAAA leader sequence found between residues 1 and 17 of mouse calreticulin. The latter sequence has been implicated in the translocation of calreticulin into the endoplasmic reticulum [36,37]. The data of Zhang et al (2006) has shown artificial Nrf2 with this N-terminal domain can anchor on endoplasmic reticulum and display less transactivation activity towards the ARE-reporter gene than wild-type Nrf2, suggesting, targeting these bZIP proteins to membranes inhibits their activity [36]. From the data of Zhang et al, we also know Nrf1 can associate with the endoplasmic reticulum and is not regulated by Keap1, whereas Nrf2 does not associate with this organelle and is regulated by Keap1. The two observations suggest these two bZIP factors mediate adaptation to redox stress in different subcellular compartments. We found the interaction between Nrf1 and MCRS2 probably played certain roles in redox stress. Our finding in this paper could explain, at least in part, the distinct regulation phenomona noted in the Nrf1 and Nrf2 KO mice.

## Conclusion

In summary, we have shown the close interaction between CNC-bZIP transcription factor Nrf1 and MCRS2. From our study, the repression of Nrf1 transactivation by MCRS2 is reported for the first time. The identification of Keap1 (Kelch-like ECH-associated protein 1) as a repressor of Nrf2 activity defines the current regulatory pathway for Nrf2 activation [38-40]. Keap1 acts by directly interacting with Nrf2 and preventing its nuclear accumulation [38-40]. In this study, we find the new repressor-MCRS2 acts as a regulator on Nrf1 transcriptional activities and the C-terminus of Nrf1 containing ZIP domain interacts with MCRS2. We show MCRS2 and Nrf1 interact through the ZIP domain of Nrf1 and the C-terminal region of MCRS2 containing coiled-coil and FHA domain. Note, the most C-terminus of Nrf1 and MCRS2 do not directly bind to each other (*row 8 *of Fig 1B and *row 7 *of Fig 1C), but it is absolutely required for this interaction. The FHA domain in MCRS2 is well conserved [41-43] and presumably mediates protein-protein interactions in some cellular processes. FHA domains are found almost exclusively in nuclear proteins, which are linked to the control of transcription, DNA repair, or cell-cycle progression [41-43]. So the interaction between Nrf1 and MCRS2 may play some important roles related to the repression of Nrf1-related transcription.

## Methods

### Plasmid construction

For the yeast two-hybrid system, Nrf1 (276–447) and its derivatives were cloned between the EcoRI and BamHI sites of pAS2-1 (CLONTECH). MCRS2 and its derivatives were cloned between the BamHI and XhoI sites of pACT2 (CLONTECH). For the bacterial protein expression construct, GST-Nrf1 derivatives were cloned between EcoRI and BamHI sites of pGEX-1 (Amersham Bioscience), while GST-MCRS2 derivatives were cloned by inserting the corresponding DNA fragment between BamHI and EcoRI sites of pGEX-5X-1 (Amersham Pharmacia Biotech). For mammalian expression and *in vitro *translation constructs, the pcDNA3HA-Nrf1 was obtained by inserting the Nrf1 cDNA between XbaI and EcoRI sites of pcDNA3-HA (Invitrogen), which had a HA epitope linked to the N-terminus of Nrf1 protein. pRK5F-MCRS2 was cloned by inserting the MCRS2 cDNA between EcoRI and XbaI sites of pRK5F (BD PharMingen), which had a FLAG epitope on the C-terminus of MCRS2 protein. Full-length MCRS2 cDNA was generated by nested PCR (polymerase chain reaction) from fetal brain cDNA library (CLONTECH) using two pairs of primers: p78-FL (5'-ATCACCAGTCTGACCTGAGCACCA-3'), p78-RL (5'-TTTTTCCAGGAGCCTGAGTTCCCA-3'), p78-N-EcoRI (5'-CCGGAATTCACATGACACGTGGCACCG-3'), P78-C-XbaI (5'-GCTCTAGACTGTGGTGTGATCTTGGC-3'). For the reporter assay, reporter tk-Luc was constructed by replacing the BglII/HindIII fragment of pGL3-basic (Promega) with the HSV-TK promoter DNA fragment from the BglII/HindIII fragment of phRL-TK (Promega). Reporter HS40-TK-Luc was formed by replacing the KpnI/BglII fragment of reporter tkLuc with the fragment of HS40 enhancer of α-globin locus control region (355 bp) from the KpnI/BglII fragment of pGL3HS40α [44] provided kindly by Dr. Shen. Reporter pGL3-control-L6 was constructed by inserting six LexA biding sites between the BglII and HindIII sites of pGL3-control reporter (Promega). LexA fusion protein expression plasmid pLexA-Nrf1 has been described before [45,46]. CMV- LacZ was constructed by inserting the LacZ gene between the SmaI and BamHI sites of pRK5F (BD PharMingen).

### Yeast two-hybrid screen

The DNA fragments encoding the C-terminal of Nrf1 (276–447), containing CNC and bZIP domain, was generated by PCR and subsequently inserted into pAS2-1 vector (Clontech) to produce GAL4DB-Nrf1 (276–447) bait. The GAL4DB-Nrf1 (276–447) bait was used to screen against human *HeLa *MATCHMAKER cDNA library (MATCHMAKER two-hybrid system; Clontech). Yeast transformation was performed using a MATCHMAKER GAL4 Two-Hybrid System 3 according to the manufacturer's instructions (Clontech). AH109 yeast strain was simultaneously cotransformed with GAL4DB-Nrf1 (276–447) bait and human *HeLa *MATCHMAKER cDNA library. The library trasformants were then selected on SD agar plate lacking leucine, tryptophan, adenine, and histidin. The plasmids extracted from blue colonies, amplified in *E. Coli *MC1061P3 strain, and retransformed with either GAL4DB-Nrf1 (276–447), GAL4DB-Lef1▲N105, or GAL4DB-lamin to test the binding specificity. The plasmids screened out from the library and confirmed for the interactions with Nrf1 (276–447) domain were then subjected to DNA sequence analysis.

### GST (Glutathione S transferase) fusion protein pull-down assay

The expression and purification of GST, GST-Nrf1 derivatives and GST-MCRS2 derivatives were all done following the previous studies [45,46]. The S^35^-labled Nrf1 and MCRS2 were made by *in vitro *transcription and translation method in reticulocyte lysate system according to the manufacturer's manual (Promega). [^35^S] methionine (Amersham Pharmacia Biotech) was transiently added in the reaction for labelling the proteins and the templates for these reactions were pcDNA3HA-Nrf1 and pRK5F-MCRS2. 40 μl of 50% GST beads containing equal amounts of each purified GST fusion protein were incubated with S^35^-labled interested protein under 4°C for 2 hours in buffer D [20 mM Hepes, 100 mM KCl, 0.2 mM EDTA, 20% glycerol, 1 mM PMSF, 1 mM dithiothreitol, 1 μg/μl BSA (bovine serum albumin, Promega)]. Then, the beads were washed with 1 ml buffer D containing 200 mM salt (20 mM Hepes, 200 mM KCl, 0.2 mM EDTA, 20% glycerol, 1 mM PMSF, 1 mM DTT) for five times. Bound proteins were eluted from the beads with 20 μl buffer D containing 0.1 M reduced form glutathione [20 mM Hepes PH 8.0, 100 mM KCl, 0.2 mM EDTA, 20% glycerol, and 0.1 M reduced form of glutathione (Sigma)] under room temperature for 20 min. Finally, the proteins in the elution supernatant were analyzed by SDS-polyacrylamide gel electrophoresis and autoradiography.

### Immunoprecipitation and western blotting

For determining the *in vivo *association of Nrf1 and MCRS2 in mammalian cells, 10 ug of indicated pcDNA3HA-Nrf1 and pRK5F-MCRS2 were co-transfected into 293 cells with 50% confluent in 10-cm dish. After transfection for 36 hours, the cells were washed once with PBS (phosphate-buffered saline), lysed in 400 μl EBC lysis buffer (420 mM NaCl, 50 mM NaF, 100 μM Na_3_VO_4_, 50 mM Tris pH8.0, 0.5% NP40, 1 mM EDTA, 5 mM MgCl_2 _and 1 mM PMSF), and rotated in eppendorff at 4°C for 30 min. Cellular debrises were pelleted by centrifugation at 18,000 × g for 15 min at 4°C. Whole cell lysates were mixed with monoclonal antibody against FLAG M2, HA, or mouse pre-immune serum, and then the immunocomplexes were mixed with 40 μl of protein G Sepharose beads [50% slurry, washed beads with NETN buffer (25 mM Tris PH8.0, 50 mM NaCl, 0.5% sodium deoxycholate, 0.5% NP40) before use, Amersham Pharmacia Biotech] and rotated in eppendorff at 4°C for 4 hours. The beads were gently washed three times with 1 ml of wash buffer (20 mM Tris PH8.0, 50 mM NaCl, 1 mM EDTA, 0.5% NP40, 5 mM MgCl_2_) followed by western blot analysis using anti-HA monoclonal antibody (Roche) and anti-FLAG M2 monoclonal antibody (Sigma).

### Cell culture, transfection and reporter gene assay

293 cells were maintained in DMEM (Dulbecco's modified Eagle's medium) containing 10% fetal bovine serum. Approximately 2.4 × 10^6 ^cells were plated on 100 mm dish 15 hours before transfection. Calcium phosphate-mediated DNA transfection was performed on 293 cells as described earlier [47]. Cell extracts were harvested 36 hours later for co-immunoprecipitation assays and western blot analysis. For the reporter gene assay, 293 cells were plated at 4 × 10^5 ^per well (6-well plate) 12~15 hours before transfection, and the cells received fresh medium 4 h before transfection. A luciferase reporter pGL3-control-L6 contaning six LexA binding sites (0.2 ug), LexA-Nrf1 fusion protein expression plasmid (0.6 ug) and MCRS2 expression plasmid (0.5 ug) were cotransfected into 293 cells. The β-galactosidase expression plasmid, CMV-LacZ (0.1 ug), was also included in this transfection as an internal control. Roughly 3 ug of total DNA/well was used in each transfection procedure. Also, the total amounts of DNA per well were kept constant after compensating with the pRK5F empty vector. After DNA transfection for 36 hours, cells were harvested and examined for luciferase activity according to the manufacturer's manual (Promega). The data were expressed by normalization with the internal control β-galactosidase activity. Each experiment was performed in triplicate and the results were presented as the means ± S.E. of at least three independent experiments.

### Immunofluorescence

293 and H1299 cells were seeded onto cover glasses at about 50% confluence 24 hours before DNA transfection. Cells were transfected with indicated plasmids expressing HA-Nrf1 and MCRS2-FLAG by calcium phosphate method. 36 hours after transfection, cells were fixed for 10 min with the fixation solution (4% paraformaldehyde prepared in PBS) and then permeabilized with 0.4% Triton X-100. The fixed sample were then incubated with anti-FLAG M2 Ab (Sigma) or anti-HA polyclonal Ab (Santa Cruz Biotechnology) for 2 hours at room temperature and washed three times with PBS followed by incubation with FITC-conjugated anti-rabbit IgG (Jackson) for HA-Nrf1 and Rhodamine-conjugated anti-mouse IgG (Jackson) for MCRS2-FLAG for 1 hours. Cells were observed under Nikon confocal laser microscopy with excitation at 488 and emission at 543 nm, respectively.

## Authors' contributions

JLW, YSL and CFH conceived this study. CFH and JLW wrote the first draft of the report and all authors contributed to the final draft. JLW, and SFW were responsible for the most research work. YJL, CCY as well as YTL managed the data. All authors read and approved the final manuscript.

## References

[B1] Hurst HC (1994). Transcription factors. 1: bZIP proteins. Protein Profile.

[B2] Pabo CO, Sauer RT (1992). Transcription factors: structural families and principles of DNA recognition. Annu Rev Biochem.

[B3] Chan JY, Cheung MC, Moi P, Chan K, Kan YW (1995). Chromosomal localization of the human NF-E2 family of bZIP transcription factors by fluorescence in situ hybridization. Hum Genet.

[B4] Blank V, Andrews NC (1997). The Maf transcription factors: regulators of differentiation. Trends Biochem Sci.

[B5] Kobayashi A, Ito E, Toki T, Kogame K, Takahashi S, Igarashi K, Hayashi N, Yamamoto M (1999). Molecular cloning and functional characterization of a new Cap'n' collar family transcription factor Nrf3. J Biol Chem.

[B6] Blackwell TK, Bowerman B, Priess JR, Weintraub H (1994). Formation of a monomeric DNA binding domain by Skn-1 bZIP and homeodomain elements. Science.

[B7] Moi P, Chan K, Asunis I, Cao A, Kan YW (1994). Isolation of NF-E2-related factor 2 (Nrf2), a NF-E2-like basic leucine zipper transcriptional activator that binds to the tandem NF-E2/AP1 repeat of the beta-globin locus control region. Proc Natl Acad Sci USA.

[B8] Chan JY, Han XL, Kan YW (1993). Cloning of Nrf1, an NF-E2-related transcription factor, by genetic selection in yeast. Proc Natl Acad Sci USA.

[B9] Andrews NC, Erdjument-Bromage H, Davidson MB, Tempst P, Orkin SH (1993). Erythroid transcription factor NF-E2 is a haematopoietic-specific basic-leucine zipper protein. Nature.

[B10] Zhang Q, Rombel I, Reddy GN, Gang JB, Shen CK (1995). Functional roles of in vivo footprinted DNA motifs within an alpha-globin enhancer. Erythroid lineage and developmental stage specificities. J Biol Chem.

[B11] Moi P, Kan YW (1990). Synergistic enhancement of globin gene expression by activator protein-1-like proteins. Proc Natl Acad Sci USA.

[B12] Philipsen S, Talbot D, Fraser P, Grosveld F (1990). The beta-globin dominant control region: hypersensitive site 2. Embo J.

[B13] Marini MG, Asunis I, Chan K, Chan JY, Kan YW, Porcu L, Cao A, Moi P (2002). Cloning MafF by recognition site screening with the NFE2 tandem repeat of HS2: analysis of its role in globin and GCSl genes regulation. Blood Cells Mol Dis.

[B14] Pondel MD, George M, Proudfoot NJ (1992). The LCR-like alpha-globin positive regulatory element functions as an enhancer in transiently transfected cells during erythroid differentiation. Nucleic Acids Res.

[B15] Rombel I, Hu KY, Zhang Q, Papayannopoulou T, Stamatoyannopoulos G, Shen CK (1995). Transcriptional activation of human adult alpha-globin genes by hypersensitive site-40 enhancer: function of nuclear factor-binding motifs occupied in erythroid cells. Proc Natl Acad Sci USA.

[B16] Husberg C, Murphy P, Bjorgo E, Kalland KH, Kolsto AB (2003). Cellular localisation and nuclear export of the human bZIP transcription factor TCF11. Biochim Biophys Acta.

[B17] Venugopal R, Jaiswal AK (1998). Nrf2 and Nrf1 in association with Jun proteins regulate antioxidant response element-mediated expression and coordinated induction of genes encoding detoxifying enzymes. Oncogene.

[B18] Venugopal R, Jaiswal AK (1996). Nrf1 and Nrf2 positively and c-Fos and Fra1 negatively regulate the human antioxidant response element-mediated expression of NAD(P)H:quinone oxidoreductase1 gene. Proc Natl Acad Sci USA.

[B19] Itoh K, Chiba T, Takahashi S, Ishii T, Igarashi K, Katoh Y, Oyake T, Hayashi N, Satoh K, Hatayama I, Yamamoto M, Nabeshima Y (1997). An Nrf2/small Maf heterodimer mediates the induction of phase II detoxifying enzyme genes through antioxidant response elements. Biochem Biophys Res Commun.

[B20] Kwong M, Kan YW, Chan JY (1999). The CNC basic leucine zipper factor, Nrf1, is essential for cell survival in response to oxidative stress-inducing agents. Role for Nrf1 in gamma-gcs(l) and gss expression in mouse fibroblasts. J Biol Chem.

[B21] Wild AC, Moinova HR, Mulcahy RT (1999). Regulation of gamma-glutamylcysteine synthetase subunit gene expression by the transcription factor Nrf2. J Biol Chem.

[B22] Marini MG, Chan K, Casula L, Kan YW, Cao A, Moi P (1997). hMAF, a small human transcription factor that heterodimerizes specifically with Nrf1 and Nrf2. J Biol Chem.

[B23] Kataoka K, Nishizawa M, Kawai S (1993). Structure-function analysis of the maf oncogene product, a member of the b-Zip protein family. J Virol.

[B24] Fujiwara KT, Kataoka K, Nishizawa M (1993). Two new members of the maf oncogene family, mafK and mafF, encode nuclear b-Zip proteins lacking putative trans-activator domain. Oncogene.

[B25] Blank V, Kim MJ, Andrews NC (1997). Human MafG is a functional partner for p45 NF-E2 in activating globin gene expression. Blood.

[B26] Bruni R, Roizman B (1998). Herpes simplex virus 1 regulatory protein ICP22 interacts with a new cell cycle-regulated factor and accumulates in a cell cycle-dependent fashion in infected cells. J Virol.

[B27] Ren Y, Busch RK, Perlaky L, Busch H (1998). The 58-kDa microspherule protein (MSP58), a nucleolar protein, interacts with nucleolar protein p120. Eur J Biochem.

[B28] Song H, Li Y, Chen G, Xing Z, Zhao J, Yokoyama KK, Li T, Zhao M (2004). Human MCRS2, a cell-cycle-dependent protein, associates with LPTS/PinX1 and reduces the telomere length. Biochem Biophys Res Commun.

[B29] Shimono K, Shimono Y, Shimokata K, Ishiguro N, Takahashi M (2005). Microspherule protein 1, Mi-2beta, and RET finger protein associate in the nucleolus and up-regulate ribosomal gene transcription. J Biol Chem.

[B30] Okumura K, Zhao M, Depinho RA, Furnari FB, Cavenee WK (2005). Cellular transformation by the MSP58 oncogene is inhibited by its physical interaction with the PTEN tumor suppressor. Proc Natl Acad Sci USA.

[B31] Lin DY, Shih HM (2002). Essential role of the 58-kDa microspherule protein in the modulation of Daxx-dependent transcriptional repression as revealed by nucleolar sequestration. J Biol Chem.

[B32] Bader AG, Schneider ML, Bister K, Hartl M (2001). TOJ3, a target of the v-Jun transcription factor, encodes a protein with transforming activity related to human microspherule protein 1 (MCRS1). Oncogene.

[B33] Chan JY, Kwong M, Lu R, Chang J, Wang B, Yen TS, Kan YW (1998). Targeted disruption of the ubiquitous CNC-bZIP transcription factor, Nrf-1, results in anemia and embryonic lethality in mice. EMBO J.

[B34] Luna L, Johnsen O, Skartlien AH, Pedeutour F, Turc-Carel C, Prydz H, Kolsto AB (1994). Molecular cloning of a putative novel human bZIP transcription factor on chromosome 17q22. Genomics.

[B35] Nioi P, Nguyen T, Sherratt PJ, Pickett CB (2005). The carboxy-terminal Neh3 domain of Nrf2 is required for transcriptional activation. Mol Cell Biol.

[B36] Zhang Y, Crouch DH, Yamamoto M, Hayes JD (2006). Negative regulation of the Nrf1 transcription factor by its N-terminal domain is independent of Keap1: Nrf1, but not Nrf2, is targeted to the endoplasmic reticulum. Biochem J.

[B37] Wang W, Chan JY (2006). Nrf1 is targeted to the endoplasmic reticulum membrane by an N-terminal transmembrane domain. Inhibition of nuclear translocation and transacting function. J Biol Chem.

[B38] Itoh K (2006). Protective mechanism against oxidative stress by Keap1/Nrf2 pathway. Seikagaku.

[B39] Dhakshinamoorthy S, Jaiswal AK (2001). Functional characterization and role of INrf2 in antioxidant response element-mediated expression and antioxidant induction of NAD(P)H:quinone oxidoreductase1 gene. Oncogene.

[B40] Itoh K, Wakabayashi N, Katoh Y, Ishii T, Igarashi K, Engel JD, Yamamoto M (1999). Keap1 represses nuclear activation of antioxidant responsive elements by Nrf2 through binding to the amino-terminal Neh2 domain. Genes Dev.

[B41] Durocher D, Jackson SP (2002). The FHA domain. FEBS Lett.

[B42] Hofmann K, Bucher P (1995). The FHA domain: a putative nuclear signalling domain found in protein kinases and transcription factors. Trends Biochem Sci.

[B43] Li J, Lee GI, Van Doren SR, Walker JC (2000). The FHA domain mediates phosphoprotein interactions. J Cell Sci.

[B44] Zhang Q, Reddy PM, Yu CY, Bastiani C, Higgs D, Stamatoyannopoulos G, Papayannopoulou T, Shen CK (1993). Transcriptional activation of human zeta 2 globin promoter by the alpha globin regulatory element (HS-40): functional role of specific nuclear factor-DNA complexes. Mol Cell Biol.

[B45] Huang CF, Wang YC, Tsao DA, Tung SF, Lin YS, Wu CW (2000). Antagonism between members of the CNC-bZIP family and the immediate-early protein IE2 of human cytomegalovirus. J Biol Chem.

[B46] Tsai HL, Kou GH, Chen SC, Wu CW, Lin YS (1996). Human cytomegalovirus immediate-early protein IE2 tethers a transcriptional repression domain to p53. J Biol Chem.

[B47] Lin YS, Green MR (1989). Similarities between prokaryotic and eukaryotic cyclic AMP-responsive promoter elements. Nature.

